# Chemokine profile in the serum of patients with leptospirosis

**DOI:** 10.3389/fcimb.2024.1484291

**Published:** 2024-10-29

**Authors:** Iago H. de Miranda Mariano, Roberta M. Blanco, Camila Eulalio de Souza, Geovanna Silva de Freitas, Paulo Lee Ho, Elizabeth A. L. Martins, Eliete C. Romero, Josefa B. da Silva

**Affiliations:** ^1^ Laboratory of Bacteriology, Butantan Institute, Sao Paulo, Brazil; ^2^ Biosciences Department, Rice University, Houston, TX, United States; ^3^ Laboratory of Bacteriology, Adolfo Lutz Institute, Sao Paulo, Brazil; ^4^ Bioindustrial Division, Butantan Institute, São Paulo, Brazil; ^5^ Laboratory of Recombinant Biological, Butantan Institute, São Paulo, Brazil

**Keywords:** chemokines, leptospirosis, diagnosis, *Leptospira*, Copenhageni serovar, protein interaction

## Abstract

**Introduction:**

Leptospirosis is a global zoonosis that affects more than one million people per year, with a lethality rate of approximately 15%. Chemokines are crucial in the immune response against *Leptospira*, recruiting leukocytes to the site of infection and regulating immune activity. In previous studies, we have shown that CCL2, CXCL5, and CCL8 are involved in the leptospirosis process, although the mechanisms are not understood.

**Methods:**

In this study, we present the frequency of *Leptospira* serovars in human samples. We then evaluated the profile of various chemokines in sera from patients diagnosed with leptospirosis, assessing the possible correlation between them. Moreover, we evaluated the changes in the chemokine profile on different days after the first symptoms. The frequency of the *Leptospira* serovars in human samples is presented.

**Results and discussion:**

The main findings were that CCL5, CXCL5, and CXCL9 are highly expressed during leptospirosis, indicating a special role of these molecules in the immunity and pathogenesis of the disease. The correlation analysis of detected chemokines CXCL11, CXCL9, CCL3, and CCL2 helps to clarify the role of each cytokine in leptospirosis. The possible use of CCL5 as a biomarker for complementary diagnosis of the disease is suggested.

## 1 Introduction

Leptospirosis is one of the most important zoonotic bacterial diseases. It is highly prevalent in the tropics, reaching over one million cases of infection per year worldwide (Costa et al., 2015; Rajapakse, 2022). Because of global climate change, heavy rains and flooding have been associated with several leptospirosis epidemics (Lau et al., 2010). Symptoms range from asymptomatic to mild febrile to severe acute infection, potentially leading to organ failure and death. Approximately 30% of cases report long-term health consequences (Costa et al., 2015; Haake and Levett, 2015).

Wild and domestic animals can be reservoir hosts, with the black rat (*Rattus rattus*) and the brown rat (*Rattus novergicus*) being the primary source of human infections (Bradley and Lockaby, 2023; Haake and Levett, 2015). *Leptospira* reproduces in the renal tubules of infected animals and is excreted via urine into the environment, contaminating water and soil; thus, environmental factors and sanitary conditions can favor its transmission, by indirect contact with soil and water or direct contact with infected animals (Bierque et al., 2020a; Browne et al., 2023; Desvars et al., 2011).

The innate immune response is the first barrier against bacteria. It involves pattern recognition receptors (PRRs) that interact with pathogen-associated molecular patterns (PAMPs) and activate the expression of specific genes (Cagliero et al., 2018). Toll-like receptors (TLRs) are PRRs that identify conserved microbial components, such as lipopolysaccharides (LPS). They are essential for the control of *Leptospira.* TLR4 is the most important LPS receptor in mice, while TLR2 is the main human receptor that responds to *Leptospira* LPS (Chassin et al., 2009; Fraga et al., 2011; Haake and Levett, 2015; Werts et al., 2001). TLRs trigger pro-inflammatory cascades, which activate transcription factors. As a result, cells express pro-inflammatory molecules such as cytokines, prostaglandins (PGs), nitric oxide (NO), and chemokines (Charo et al., 2019; Kawai and Akira, 2007; Santecchia et al., 2020).

Chemokines partake in the immune response against bacterial infections such as pneumonia (Standiford et al., 1996), tuberculosis (Domingo-Gonzalez et al., 2016), and leptospirosis (Silva et al., 2019). They serve as a chemoattractant, recruiting leukocytes to the site of damage/infection (Cagliero et al., 2018). CCL2 is one example of chemokine from the CC subfamily produced by many cell types, the most important of which are monocytes and macrophages (Bachelerie et al., 2014; Bose and Cho, 2013). Cytokines control the migration of monocytes and macrophages to the target tissue, providing local defense and repair of tissue damage (Deshmane et al., 2009; Uhlén et al., 2015).

Other chemokines of the CC subfamily have been extensively studied. CCL5 is a potent chemoattractant of lymphocytes and monocytes that acts via CCR1 and CCR5, contributing to the recruitment of T cells and macrophages (Araujo et al., 2018). It has been associated with cancer, atherosclerosis, inflammatory bowel diseases, and other diseases (Zeng et al., 2022). Moreover, CCL3 is produced by lymphocytes and fibroblasts, recruiting lymphocytes to sites of infection and CD8+ T cells to lymph nodes (Castellino et al., 2006). Similarly, CCL28, expressed by epithelial cells in the intestine, lung, and salivary glands, drives the migration of T and B lymphocytes to the mucosa^26^.

The CXC subfamily of chemokines also acts as a chemoattractant for immune cells (Bachelerie et al., 2014). CXCL5 recruits neutrophils, the most abundant type of leukocyte, to sites of inflammation and contributes to the Th17 lymphocyte response (Disteldorf et al., 2015). Similarly, CXCL9 mediates lymphocytic infiltration (Tokunaga et al., 2018), and CXCL11 is correlated with CD8+ T-cell infiltration (Li et al., 2022).

There are several knowledge gaps regarding the role of chemokines in *Leptospira* infection. Previous findings from our group have shown that CCL2 does not interfere with the phagocytosis of *Leptospira* by spleen cells in either susceptible or resistant mice. However, CCL2 has been shown to have a potential to modulate other chemokines involved in the immune response (Silva et al., 2019).

Here, we report the chemokine profile found in the serum of leptospirosis patients and the potential for protein–protein interactions by STRING analysis. We found that CCL5, CXCL9, and CXCL5 were the most expressed chemokines and are probably overexpressed during infection. These findings corroborate our data showing an increase of chemokines in the spleen and lung of Balb/c (resistant strain) infected with pathogenic *Leptospira* (Domingos et al., 2017). It seems that these chemokines play a key role in the host defense and their resistance to a more severe *Leptospira* infection.

## 2 Material and methods

### 2.1 Characterization of the serum samples in the study

The evaluation of the chemokine profile in the serum of patients with leptospirosis, in the acute or convalescent phase (total *n* = 103), was carried out with isolated or paired samples. For paired samples, the microscopic agglutination test (MAT) was negative for the first sample. Samples from healthy individuals or patients with other febrile illnesses for whom the MAT results were negative were used as control (*n* = 5). The sera were obtained from the bank of samples stored (−80˚C) at the Adolfo Lutz Institute after MAT was carried out for the routine leptospirosis diagnosis. Usage was approved by the Research Ethics Committee of the Adolfo Lutz Institute and the National Research Ethics Committee (CONEP) of the Brazil’s Ministry of Health in accordance with protocol no. 31938820.3.1001.0059.

### 2.2 Microscopic agglutination test

MAT was carried out at the Adolfo Lutz Institute using the standard procedure (Faine, 1999), with serovars representative of different serogroups known to be prevalent in São Paulo, Brazil (Blanco and Romero, 2015): Australis, Autumnalis, Bataviae, Canicola, Castellonis, Copenhageni, Cynopteri, Djasiman, Grippotyphosa, Hardjo, Hebdomadis, Icterohaemorrhagiae, Javanica, Panama, Patoc, Pomona, Pyrogenes, Sejroe, Tarassovi, and Wolfii. The strains were obtained from the National Reference Center for Leptospirosis at Fiocruz-RJ and from Adolfo Lutz Institute-SP, Brazil. The strains were cultured and maintained in liquid media EMJH (Ellinghausen–McCullough–Jonson–Harris) for 7 days at 30°C. Titers equal to or greater than 1:200 were considered positive. The probable infecting serogroup was defined as the serogroup with the maximum titer directed against a single serovar. A confirmed case of MAT was defined as seroconversion between samples from the acute phase and the convalescent phase.

### 2.3 Determination of chemokines by ELISA and protein interaction by database analysis

The chemokine profile in the patients’ serum was determined by ELISA using commercial kits (R&D Systems): CCL2/MCP1, CCL3/MIP-alpha, CCL5/RANTES, CCL28/MEC, CXCL5, CXCL9, CXCL10/IP-10, and CXCL11. Statistically significant chemokine expression variations were analyzed considering functional interaction networks by the STRING database (https://string-db.org), which allows a deep analysis on protein interaction based on many parameters, such as genomic neighborhood, functional pathway, experimental evidence, co-expression, and citation among others. Our basic settings were adjusted for full string networks; no more 5.0 interactions; interaction score threshold of highest confidence, 0.9; active interaction sources: experiments, co-occurrence, and co-expression.

### 2.4 Statistical analysis

Analysis by Student’s *t*-tests or two-way ANOVA alpha and Tukey multiple comparisons were applied to assess significant differences (*p* ≤ 0.05) of the chemokines in leptospirosis patient samples. Statistical analysis and data plotting were carried out using Prism software (GraphPad).

## 3 Results

### 3.1 Characterization of human serum samples: MAT titers and frequency of specific serovars in the infection

**Figure 1:**
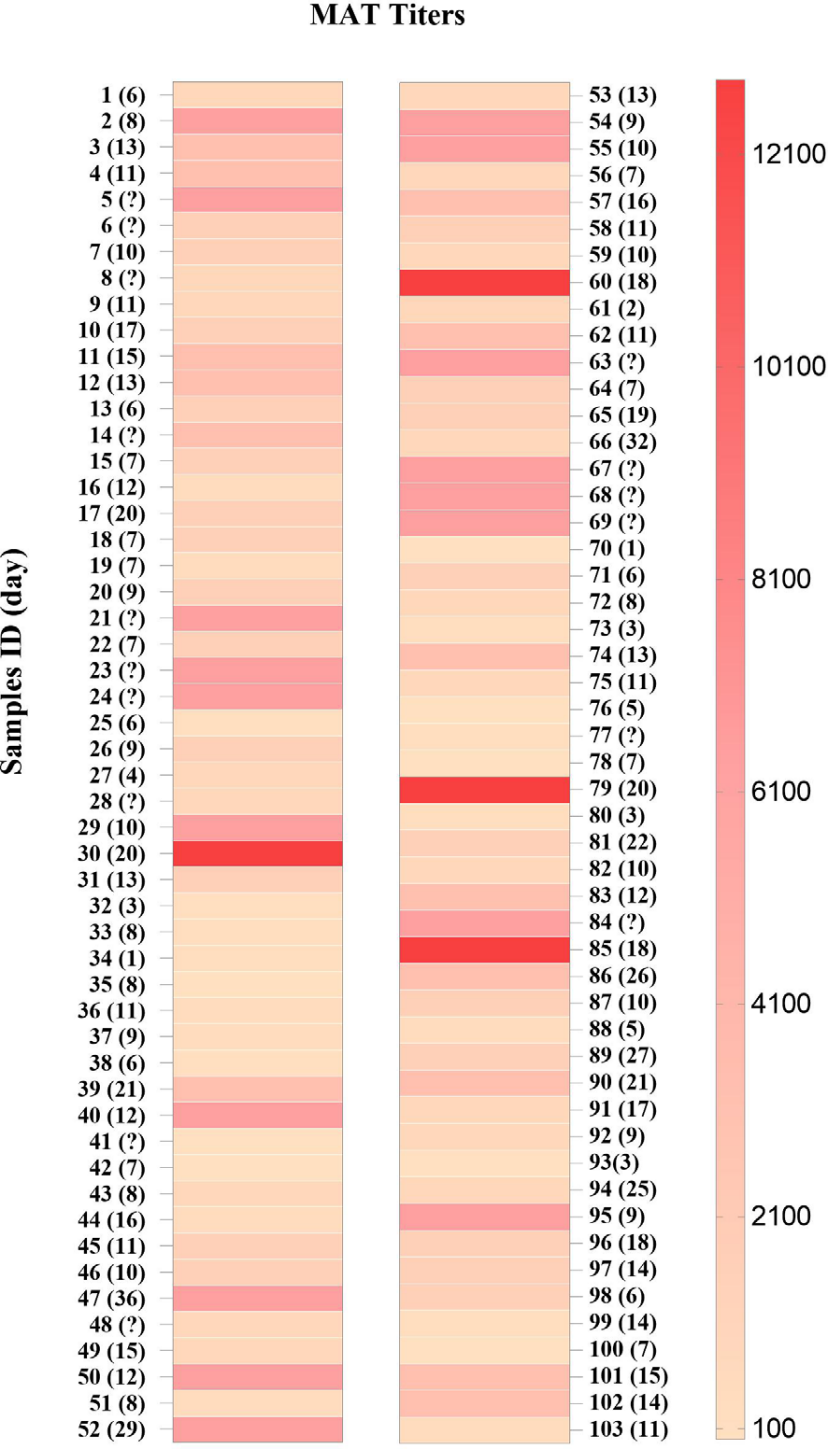
MAT titers in sera from patients with leptospirosis (*n* = 103). Samples are numbered sequentially. The days after the first symptoms are shown in parentheses and (?) indicates no data.

**Figure 2:**
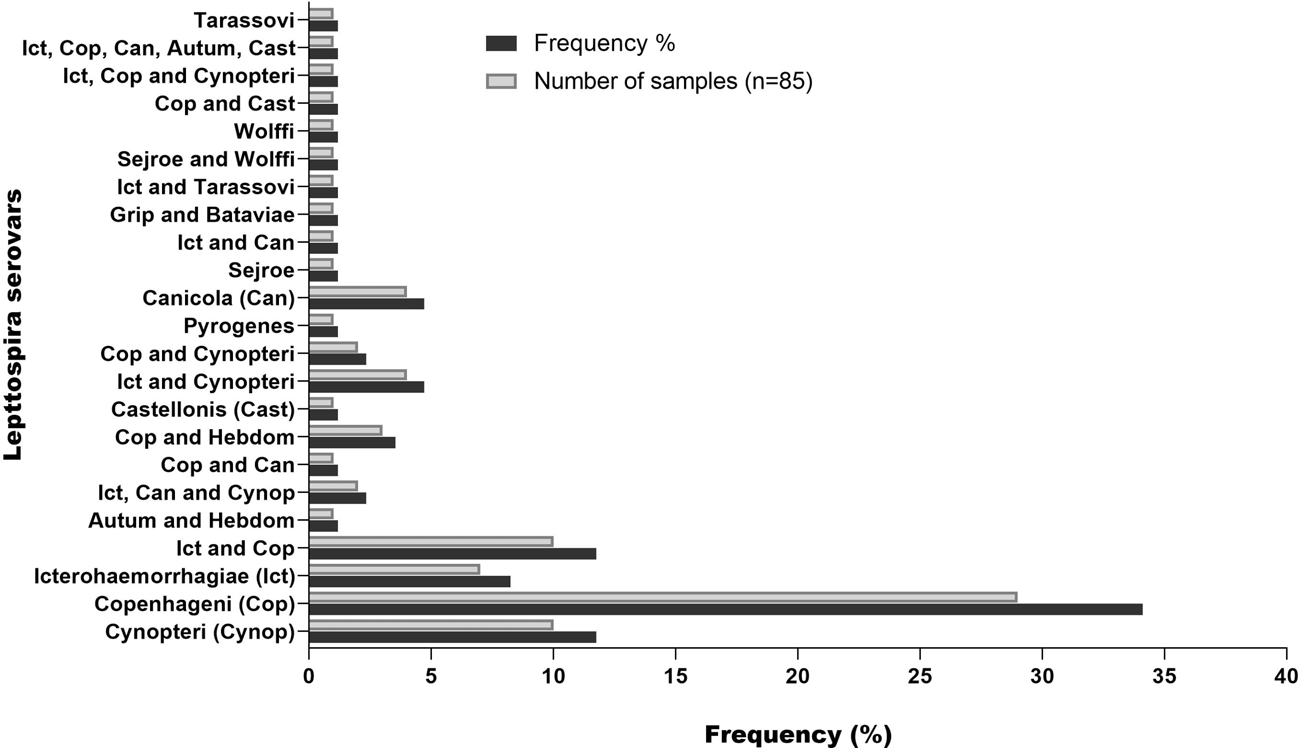
Frequency of serovar detected by MAT in serum samples from patients with leptospirosis. Second samples from the same patient were included when MAT indicated a different serovar from the first samples analyzed.

A total of 103 serum samples from 85 individuals with leptospirosis confirmed by MAT (**Figure 1**) were classified based on the serovars (**Figure 2**). The most frequent serovar in samples were Copenhageni (Cop; <30%) (**Figure 2**). Second samples from the same patient were included when MAT indicated a different serovar from the first samples analyzed. Our data are consistent with public data on the prevalence of serovars in Brazil with Copenhageni and Icterohaemorrhagiae being the most common serovars (Arent et al., 2023; Browne et al., 2023). Both have been the most commonly diagnosed since 1999 (Ko et al., 1999).

### 3.2 Chemokine profile in the serum of patients with leptospirosis

**Figure 3:**
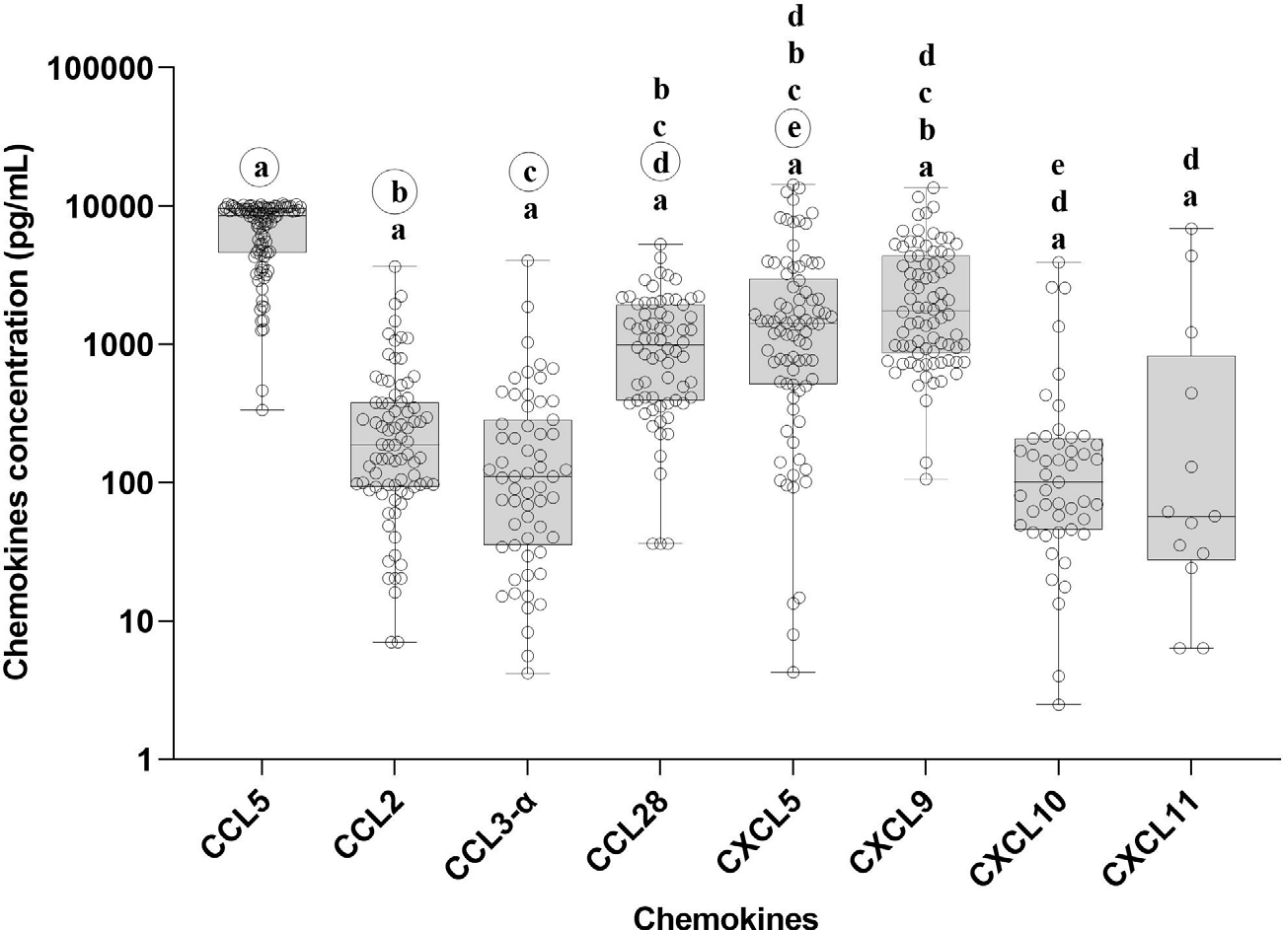
Chemokine profile in the serum of leptospirosis patients—analysis among groups (*n* = 103). The significance of variation of concentration of chemokines was analyzed by Tukey’s comparison test. Significances are represented by letters. Letters inside circles are the references for comparison with other groups. All the data on statistical comparison among chemokines are in **Supplementary Table S1**.

**Figure 4:**
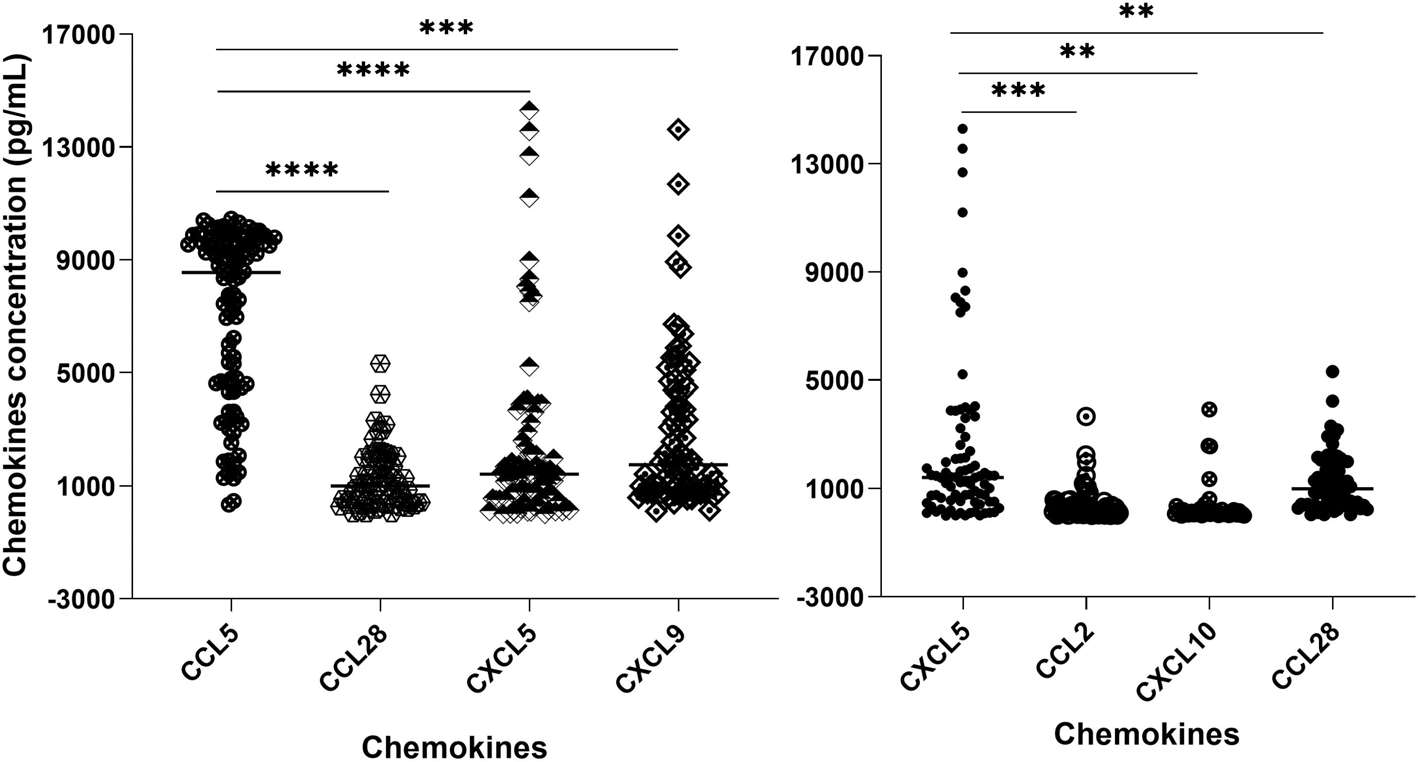
Chemokine profile in the serum of leptospirosis patients (*n* = 103)—analysis in each sample and by groups. The significance of variation was analyzed by Tukey’s comparison test, highlighting the expressed chemokines CCL5 and CXCL5 per sample and among the groups. All the data on statistical comparison among chemokines are shown in **Supplementary Table S1**. The symbol ** refers to *p* < 0.05 and the symbol *** and **** refers to *p* < 0.0001.

We measured the chemokine profile of 103 serum samples with leptospirosis confirmed by MAT. Using ELISA, we determine the main chemokines increased in human serum in response to infection by *Leptospira* spp. We compared the overall levels of chemokine in serum sample groups (**Figure 3**; **Supplementary Table S1**) and the levels of each chemokine in each patient (**Figure 4**; **Supplementary Table S1**), analyzing by two-way ANOVA alpha, considering significance *p* ≤ 0.5. In general, CCL5, CXCL5, and CXCL9 were the most expressed chemokines (**Figures 3**, **4**). CCL5 was highly expressed in the majority of patients (**Figures 3**, **4**), and it was significantly higher when compared to CXCL5, CCL28, and CXCL9 (**Figure 4**).

Our data showed a higher level of CXCL5 compared to CCL2, CCL28, and CXCL10 (**Figure 4**), while CCL28 was significantly higher than CXCL11. On the other hand, CCL2, CCL3-α, CXCL10, and CXCL11 showed lower expression levels (**Figure 3**).

We analyzed the possible correlation of the main chemokines expressed, CCL5, CXCL5, and CXCL9, in the days following the symptoms, highlighting that only CCL5 showed an increasing concentration up to the 30th day (**Supplementary Table S3**).

### 3.3 Pearson’s correlation in the chemokine profile

**Figure 5:**
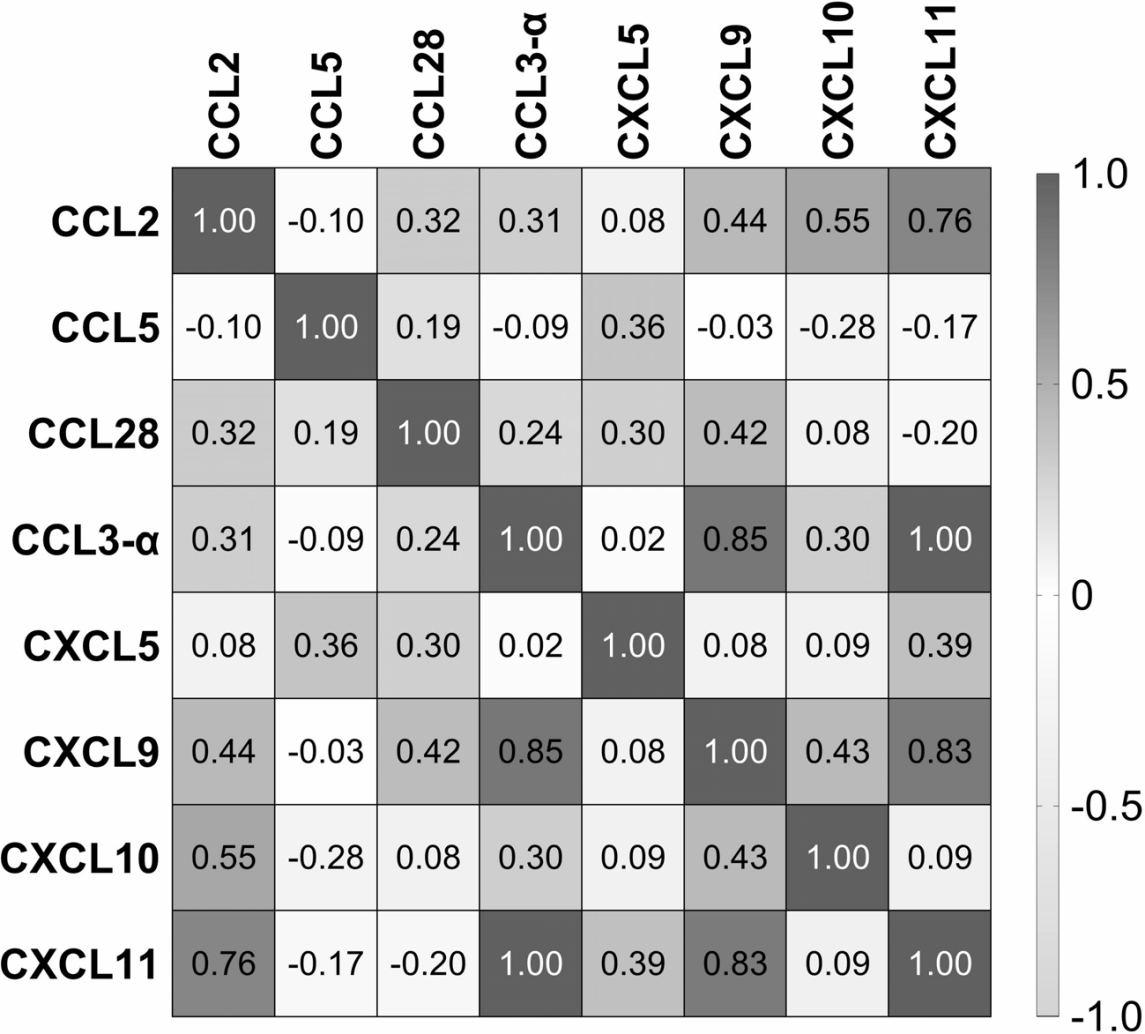
Correlation of chemokines in the serum of patients with leptospirosis (*n* = 103). Numbers inside squares are the values of Pearson’s correlation (*r*).

We used Pearson’s correlation analysis to determine the possible linear correlation between the chemokines expressed during *Leptospira* infection. The results showed a strong correlation between CXCL11, CXCL9, and CCL3 (**Figure 5**). In addition, CCL2 had a strong correlation with CXCL11. There was a moderate correlation between CXCL10 and CXCL9 and between CXCL9 and CCL28 (**Figure 5**). On the other hand, CCL5, which was the most expressed chemokine (**Figures 2**, **4**), and CXCL5 showed weak or no correlation with the other chemokines (**Figure 5**).

### 3.4 Chemokine profile on different days after the first symptoms

**Figure 6:**
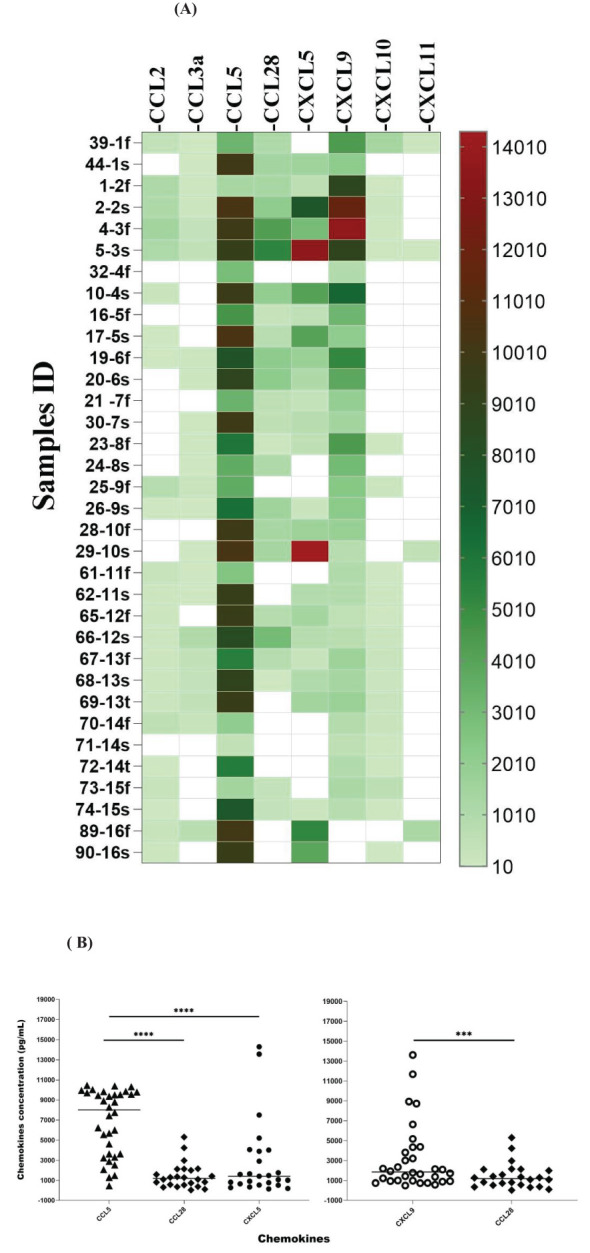
Chemokine profile in paired samples from the same patient collected on different days after symptoms (f: first sample; s: second sample; t: third sample). Analyses of three consecutive samples (f, s, and t) from two patients are shown (samples 67, 68, and 69 and samples 70, 71, and 72). **(A)** The color scale represents concentration (pg/mL) measured by ELISA. White means chemokines are not detected. **(B)** Significance is presented when there are differences for row and column data, as shown in **Supplementary Table S2**. The statistical significance in the comparison of other chemokines is shown in **Supplementary Table S2**. The symbol *** refers to *p* < 0.001 and **** refers to *p* < 0.0001. The significance of the data was analyzed using Tukey’s comparison test.

The expression of chemokines can change over the course of the disease, modulating the immune response in different ways (Shetty et al., 2021). We evaluated the chemokine profile in the serum of 16 patients at two different times after the first symptoms to check for possible variation over time. CCL5 was detected in 100% of the samples, and almost all of the second samples showed an increase in expression (**Figures 6A, B**). CXCL9, one of the most expressed chemokines (**Figure 3**), was detected in approximately 95% of the serum samples (**Figure 6A**), suggesting that it is important in the host response against *Leptospira*. Furthermore, we detected CXCL5 in 81% of the patients (13) and an increasing concentration in the second sample of 10 of these patients (**Figures 6A, B**).

CCL2 was detected in the samples of 14 patients, and 2 showed elevated levels; CCL3-α was present in the samples of 12 patients, and 3 showed an increase in the second sample; CCL28 was detected in 13 patients, with 3 increasing in the second sample (**Figure 6A**); CXCL10 was present in the samples of 10 patients with an increase in the second time point; and CXCL11 was detected in 4 samples, and 1 showed elevated levels (**Figure 6A**).

Overall, our data suggest that these chemokines are expressed in response to leptospirosis and their expression is modulated along with the development of the disease. Moreover, CCL5, CXCL5 (**Figure 6B**), and CXCL9 are the most expressed chemokines and appear to be upregulated over time (**Figure 6A**).

### 3.5 Protein–protein interaction analysis

**Figure 7:**
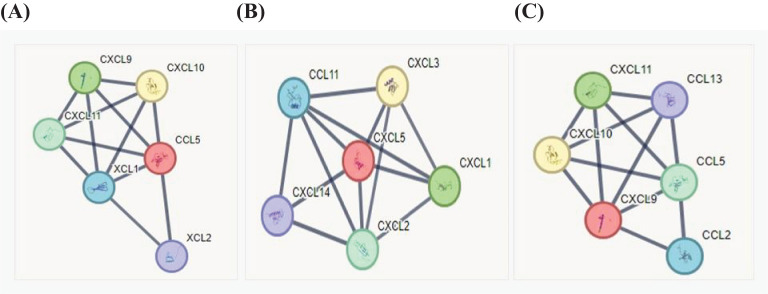
Analyses of chemokine interaction using the STRING database. The inputs for analysis were the main chemokines detected in the samples of patients with leptospirosis. **(A)** CCL5, **(B)** CXCL5, and **(C)** CXCL9 for the interaction network: the black line means that the confidence of the scoring edges is greater than 0.9.

We analyzed the protein–protein interaction using STRING (https://string-db.org), which allows for an in-depth analysis on protein interaction based on many parameters, such as genomic neighborhood, functional pathway, experimental evidence, co-expression, and citation. Statistically significant chemokine expression variations were analyzed considering functional interaction networks by the STRING database adjusting the parameters for full string networks; no more 5.0 interactions; interaction score threshold of highest confidence, 0.9; active interaction sources: experiments, co-occurrence, and co-expression. We investigated the interactions of the most expressed chemokines: CCL5 (https://version-12-0.string-db.org/cgi/network?networkId=bq7cxiSpA2Md), CXCL5 (https://version-12-0.string-db.org/cgi/network?networkId=bCG2Q5827aRn), and CXCL9 (https://version-12-0.string-db.org/cgi/network?networkId=bNqrw2vkCAXV). Our data revealed that CCL5 interacts with CXCL9, CXCL10, and CXCL11 (**Figures 7A, C**). Interestingly, all these chemokines are ligands to the CXCR3 receptor, which might be related to leptospirosis resistance. It has been described that CXCL5 leads to CXCL3, CXCL1, and CXCL14 expression, which are all potent chemoattractants for neutrophils (Zhou et al., 2023), indicating the occurrence of this mechanism on leptospirosis immune response (**Figure 7B**).

## 4 Discussion

Chemokines play a major role in the host’s immune defense (Haake and Levett, 2015). Their expression is strongly related to susceptibility to leptospirosis and organ damage (Domingos et al., 2017; Silva et al., 2020). We analyzed the profile of chemokines in patients diagnosed with leptospirosis to better understand the immune response in humans. We found that CCL5 was the most expressed chemokine in humans and was upregulated over time. CCL5 promotes early protection against *Leptospira* spp. by preventing cytokine storms in the immune response (Vesosky et al., 2010). We have previously shown that resistant and partially resistant mouse strains show an increase in CCL5 24 h after *Leptospira* infection (Arent et al., 2023; Domingos et al., 2017). The CCL5 levels seen in sera from leptospirosis patients suggest that the same mechanisms occur in humans, since CCL5 is important for regulating inflammation. NK and T helper cells unable to produce CCL5 are less efficient at recruiting DCs and cytotoxic T lymphocytes (Seo et al., 2020).

Regarding CXCL9, we showed that mice susceptible to leptospirosis (HeJ) had lower levels of this chemokine when compared to resistant mice (Balb/c) (Domingos et al., 2017), suggesting that it plays an important role in the host response. CXCL9 is upregulated during infection with pathogenic *Leptospira* and binds to the CXCR3 receptor, which is expressed at all stages of CD4 T-cell development (Rabin et al., 2003; Shetty et al., 2021; Tannenbaum et al., 1998). The CXCR3 axis regulates the differentiation of naïve T cells into helpers 1 and drives migration to their target sites. In *Salmonella* infection, the CXCR3 axis controls dissemination of bacteria. In addition, the expression of CXCR3 on CD8+ T cells increases their antibacterial activity (Chami et al., 2017; Oghumu et al., 2015; Ridley and Dyer, 2022). CXCR3 has been associated with antibacterial activity against *Streptococcus pyogenes*, contributing to the antimicrobial protection of the gut (Egesten et al., 2007; Reid-Yu et al., 2015).

Recombinant CXCL9 has been correlated as being 10 times more potent as an antibacterial than CXCL11 and CXCL10 (Reid-Yu et al., 2015). Moreover, CCL5 and CXCL9 together have been correlated with CD8+ T-cell infiltration (Dangaj et al., 2019), indicating an important role in the control of leptospirosis.

CXCL5 is associated with neutrophil recruitment (Persson et al., 2003). The increased concentration of this molecule detected in our study in the serum of patients with leptospirosis is in line with our previous *in vivo* studies, which demonstrated that CXCL5 is inhibited in susceptible mice and increased in resistant ones, implying an importance for the host response (Domingos et al., 2017). In addition, *in vitro* experiments showed that raw macrophages treated with CCL2 had a reduction in CXCL5 expression, implying a negative correlation between them (Silva et al., 2019).

The main findings in our study were that CCL5, CXCL5, and CXCL9 are highly expressed in human leptospirosis disease. This study with human sera combined with our animal studies indicates that these chemokines play an important role in leptospirosis immunity. However, further studies are needed to clarify the role of these molecules in the effective immune response. We suggest that CCL5 could be used as a biomarker for the complementary diagnosis of the disease, since its expression was the most prominently observed in patients with leptospirosis.

## Data Availability

The original contributions presented in the study are included in the article/**Supplementary Material**. Further inquiries can be directed to the corresponding author.
